# Human transmissible spongiform encephalopathies in eleven countries: diagnostic pattern across time, 1993–2002

**DOI:** 10.1186/1471-2458-6-278

**Published:** 2006-11-10

**Authors:** Jesús de Pedro-Cuesta, Markus Glatzel, Javier Almazán, Katharina Stoeck, Vittorio Mellina, Maria Puopolo, Maurizio Pocchiari, Inga Zerr, Hans A Kretszchmar, Jean-Philippe Brandel, Nicole Delasnerie-Lauprêtre, Annick Alpérovitch, Cornelia Van Duijn, Pascual Sanchez-Juan, Steven Collins, Victoria Lewis, Gerard H Jansen, Michael B Coulthart, Ellen Gelpi, Herbert Budka, Eva Mitrova

**Affiliations:** 1 Instituto de Salud Carlos III, Centro Nacional de Epidemiologia, Departamento de Epidemiologia Aplicada, Calle Sinesio Delgado 6, 28029, Madrid, Spain; 2 Institute of Neuropathology, University Medical Center Hamburg-Eppendorf, Martinistraße 52, D-20246 Hamburg, Germany; 3 Institute of Neuropathology, University Hospital Zurich, Switzerland; 4 Registry of Creutzfeldt-Jakob disease, -Department of Cell. Biology and Neurosciences, Istituto Superiore di Sanita, Viale Regina Elena 299, 00161 Rome, Italy; 5 Department of Neurology, Georg-August-Universität Göttingen, Robert-Koch Strasse 40, 37075 Gottingen, Germany; 6 Department. of Neuropathology, Ludwig-Maximilian University, Munich, Germany; 7 U.708 INSERM, Hopital de la Salpetriere, 75651 Paris, Cedex 13, France; 8 Department of Epidemiology and Biostatistics, Erasmus MC, PO Box 1738, 3000 DR Rotterdam, The Netherlands; 9 Australian National Creutzfeldt-Jakob disease Registry, Department of Pathology, The University of Melbourne, Parkville, Australia; 10 CJD Surveillance System, Division of Host Genetics and Prion Diseases, Public Health Agency of Canada, LCDC Building, AL 0601E2, Tunney's Pasture, Ottawa, Ontario, K1A 0L2, Canada; 11 Institute of Neurology, Medical University of Vienna, and Austrian Reference Centre for Human Prion Diseases, AKH 4J, A-1097 Vienna, Austria; 12 Research base of Slovak Medical University, Bratislava, Slovakia

## Abstract

**Background:**

The objective of this study was to describe the diagnostic panorama of human transmissible spongiform encephalopathies across 11 countries.

**Methods:**

From data collected for surveillance purposes, we describe annual proportions of deaths due to different human transmissible spongiform encephalopathies in eleven EUROCJD-consortium countries over the period 1993–2002, as well as variations in the use of diagnostic tests. Using logistic models we quantified international differences and changes across time.

**Results:**

In general, pre-mortem use of diagnostic investigations increased with time. International differences in pathological confirmation of sporadic Creutzfeldt-Jakob disease, stable over time, were evident. Compared to their counterparts, some countries displayed remarkable patterns, such as: 1) the high proportion, increasing with time, of variant Creutzfeldt-Jakob disease in the United Kingdom, (OR 607.99 95%CI 84.72–4363.40), and France (OR 18.35, 95%CI 2.20–152.83); 2) high, decreasing proportions of iatrogenic Creutzfeldt-Jakob disease in France, (OR 5.81 95%CI 4.09–8.24), and the United Kingdom, (OR 1.54 95%CI 1.03–2.30); and, 3) high and stable ratios of genetic forms in Slovakia (OR 21.82 95%CI 12.42–38.33) and Italy (OR 2.12 95%CI 1.69–2.68).

**Conclusion:**

Considerable international variation in aetiological subtypes of human transmissible spongiform encephalopathies was evident over the observation period. With the exception of variant Creutzfeldt-Jakob disease and iatrogenic Creutzfeldt-Jakob disease in France and the United Kingdom, these differences persisted across time.

## Background

Human Transmissible Spongiform Encephalopathies (HTSE) constitute a group of rare, fatal central nervous system disorders [1]. A general characteristic of HTSE is deposition of a pathological isoform (termed prion protein; PrPSc) of the normal cellular prion protein (PrPC). Diverse aetiological factors and pathogenic mechanisms underlie the development of HTSE. The sporadic form of Creutzfeldt-Jakob disease (sCJD), which is of unknown aetiology, represents the most common form of HTSE, with an estimated yearly incidence of 1–1.5 per million population. An inherited form, caused by mutations in the gene encoding PrPC (*PRNP*) is known as familial or genetic Creutzfeldt-Jakob disease (gCJD), or genetic HTSE (gHTSE) when other recognized entities are included. Acquired forms of HTSE, such as iatrogenic Creutzfeldt-Jakob disease (iCJD) or variant Creutzfeldt-Jakob disease (vCJD), are caused by exposure to infectious prions, be it through contaminated medical products (iCJD) or uptake of bovine spongiform encephalopathy (BSE) prions, (vCJD) [2,4]. The period 1993–2002 witnessed an historically exceptional situation, inasmuch as eleven countries (the EUROCJD consortium) conducted co-ordinated epidemiological HTSE surveillance, using harmonised methods against a rapidly changing medical HTSE background. New entities, such as vCJD, were identified and reported in a number of countries [5]. Diagnostic tests were developed and case definitions modified [6,7] and infrequent forms of HTSE were documented [8]. The descriptive epidemiology of HTSE in these populations, plus time intervals and details about HTSE survival, iCJD and gHTSE have recently been reported [9-12].

Taking into account that individual countries are facing country-specific risk factors, the need for a comparison of national registries and temporal analysis of national surveillance data is imperative, highlighted by recent research indicating that the incidence of HTSE by aetiological subtype varies widely geographically [9,13,14]. The fact that BSE exposure may result in sCJD-like phenotypes, as suggested by experiments performed on rodents, further underlines the need for in-depth temporal analysis of HTSE [15,16]. We analysed variations in defined epidemiological parameters in the EUROCJD-consortium HTSE cohort over a ten-year period. Through this analysis we have been able to describe variations in the annual national panorama of investigations used to evaluate patients and in aetiological HTSE subtypes over a defined timeframe, both overall and by country.

## Methods

Cases included in this study were those registered in the national databases of constituent countries of the European Union's and Allied countries' prospective CJD surveillance programme (EUROCJD). National registers started collecting prospective data in 1979 in Slovakia, 1990 in the UK, 1992 in France, and 1993 in Germany, Italy, The Netherlands and Australia. Spain followed in 1995, as did Austria and Switzerland in 1996, and Canada in 1997. In 1993, a standardised protocol was introduced for diagnosing cases for epidemiological surveillance purposes. After the introduction of a diagnostic test based on detection of 14.3.3 protein in cerebrospinal fluid (CSF) in 1997, this protocol was updated in 1999. Diagnostic categories were based on application of shared specific diagnostic criteria for HTSE [17,18] adapted from those originally proposed by Masters and colleagues [19], updating in 1998 for surveillance purposes diagnostic criteria for sCJD [7,9,20]. The study included all patients with a diagnosis of probable or definite sCJD, gHTSE, iCJD or vCJD, who died in the period 1993–2002 and were reported to their respective national surveillance centres. All information was centralised in Rotterdam.

The patients from each country' included in the study is summarised in Table 1. The pooled dataset comprised 4441 patients: sCJD n = 3720 (2461 definite, 1269 probable); gHTSE n = 455; iCJD n = 138; and vCJD n = 128. Iatrogenic forms were due to dura mater grafts (n = 32), growth hormone (n = 105) and corneal transplantation (n = 1).

Whenever possible, all diagnostic investigations were reviewed by a member of the surveillance system. Information on electroencephalogram (EEG) records was available in 3825 cases. Typical periodic sharp and slow wave complexes (PSWCs) were deemed to be present when periodic complexes had a bi- or triphasic morphology, lasted 100–600 ms and were found to be synchronous, generalised or lateralised on the EEG and persist for at least 10 seconds [21]. Cerebral magnetic resonance imaging (MRI) data was available in 2013 individuals, and was considered positive for sCJD when high signal changes were present in striatum [22]. Analysis of results according to specific pulse sequences used for MRI studies (such as diffusion weighted imaging) was not undertaken. Information on 14.3.3 protein in CSF was available in 2890 cases, with 14.3.3 Western blotting was performed in single, national centres within each country with considerable expertise in the assay [4,7]. Genetic analysis was performed by sequencing the entire open reading frame of PRNP and was available for 2967 patients.

While time trends of diagnostic patterns of a group of entities may refer to a varied type of approaches, in this paper we describe distributions by year of death and proportions of aetiological HTSE type or diagnostic test used in clinical workup. Changes across time in HTSE incidence or mortality have been reported in prior papers [9,18]. Therefore, differences between individual countries and changes across time were explored here by combining national HTSE death ratios for different diagnostic categories: 1) for each aetiological subtype of disorder; 2) for sCJD, by pathological or non-diagnostic confirmation, i.e., probable/definite ratios; and, 3) for different entities by available specific diagnostic CSF or genotype information. The statistical significance of the variation in distributions was assessed using chi-square statistics. Logistic regression was used to calculate odds ratios (OR) for each country compared to the other 10, with adjustment for age at death and year of death: 1) for specific aetiological subtypes of disorder versus all remaining HTSE; and 2) for definite versus probable sCJD. For the purposes of comparability, the vCJD death ratios for the remaining 9 countries were taken as reference for France and the UK alike. OR time trends were ascertained by introducing time as a continuous independent variable in the models. In order to judge the additional contributions made by EEG patterns and presence of 14.3.3 protein in CSF to sCJD diagnosis, and by identification of mutations in PRNP to any gHTSE diagnosis, we calculated the annual proportions of positive findings in each of these auxiliary tests in cases where the corresponding findings in EEG, 14.3.3 protein test, or family history of HTSE were absence of PSWCS or negative.

The information used in the paper is grouped and mortality grouped data for each country is publicly available at the web at the CJD surveillance unit in Edinburgh. The study underwent evaluation by the Medical Committee on Ethics of the Rotterdam Academic Hospital, MEC 127.071/1993/71, and by the Carlos III Institute of Health Committee on Bioethics, report number Pub-102/06.

## Results

**Figure 1:**
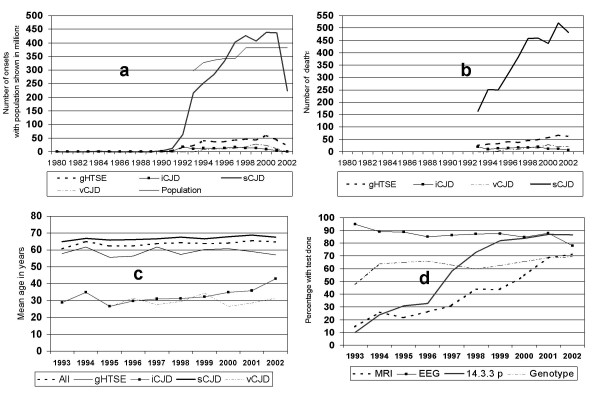
Variation across time for four major HTSE groups in: a) annual number of cases by year of clinical onset, with study population shown in millions; b) annual number of cases by year of death; c) annual average age at death and; d) proportion of cases, all entities, for whom diagnostic data were available in terms of EEG, 14.3.3 test in CSF, MRI and genotyping, by year of death

Shown in Figure 1 are graphs depicting the population under surveillance (Figure 1-a) and annual data in the EUROCJD consortium countries for major HTSE categories in terms of number of clinical onsets (Figure 1-a), number of deaths (Figure 1-b), average age at death (Figure 1-c), as well as the proportion of HTSE deaths for which EEG, CSF 14.3.3, cerebral MRI and genotyping results were available (Figure 1-d). The most remarkable general feature was that at no period was the death or incidence rate stable from year to year. For most entities, the highest annual number of HTSE onsets occurred early in the second half of the observation period (Figure 1-a). The increase in onsets from 1993 to 1998 was mainly in evidence for vCJD (0 to 18 cases), gHTSE (22 to 47 cases) and sCJD (216 to 426 cases). In addition, discordance between onset and death was observable for both sCJD and other entities at the end of the study period, a finding consistent with selecting cases by death up to 2002, for inclusion in a database. While a statistically significant linear trend for increased average age at death was observed for sCJD (0.30 95%CI 0.14–0.46 years per year), and iCJD (1.15 95%CI 0.26–1.95 years per year), no such trend was suggested for gHTSE or vCJD (Figure 1-c).

The use of ancillary tests, such as MRI, 14.3.3 or genetic analysis, in diagnosis of HTSE (Figure 1-d) increased with time up to 2002. Overall, the most frequently used investigation was the EEG, yet in recent years the use of the CSF 14.3.3 protein analysis increased significantly, attaining levels comparable to EEG analyses in 2000. While rising time trends were seen for 14.3.3 tests and MRI, stable values for PRNP genotyping and decreasing figures for EEG examination were in evidence.

**Figure 2:**
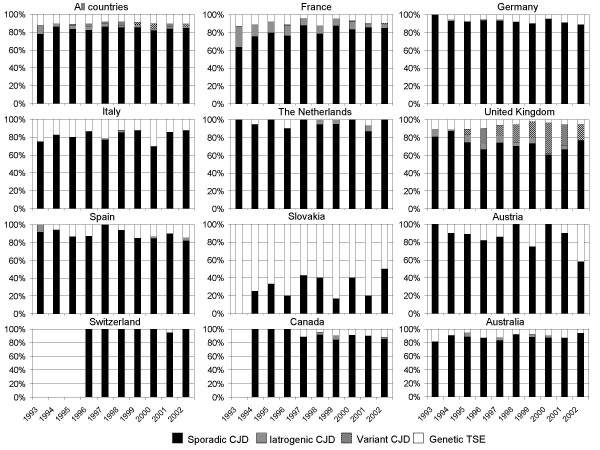
Annual distribution by type of HTSE in eleven countries. Different study intervals for the period, 1993–2002.

Annual distribution by HTSE type (Figure 2) changed significantly, both overall (p < 0.001) and in the UK, p < 0.001, and France p = 0.009 (Pearson χ^2^). Furthermore, country- specific patterns were observable, i.e., an increase in vCJD in the UK and a decrease in iCJD in France. In the comparative inter-country analysis focusing on specific diagnostic groups, remarkable differences in magnitude and linear time trends were found. Of interest were: 1) an increase over time in vCJD in the UK (OR 607.99 95%CI 84.72–4363.40; time trend OR 1.21, 95%CI 1.11–1.31), and France (OR 18.35, 95%CI 2.20–152.83; time trend OR 1.64 95%CI 1.02–2.62), as against other countries; 2) a decrease over time in iCJD in France, OR 5.81 95%CI 4.09–8.24; time trend OR 0.84, 95%CI 0.80–0.90), and in the UK, (OR 1.54 95%CI 1.03–2.30, time trend OR 0.85 95%CI 0.79–0.90); and 3) a high proportion of gHTSE in Slovakia, (OR 21.82 95%CI 12.42–38.33) and Italy, (OR 2.12 95%CI 1.69–2.68), with stable time trends in both countries, (OR 0.99 95%CI 0.95–1.03).

**Figure 3:**
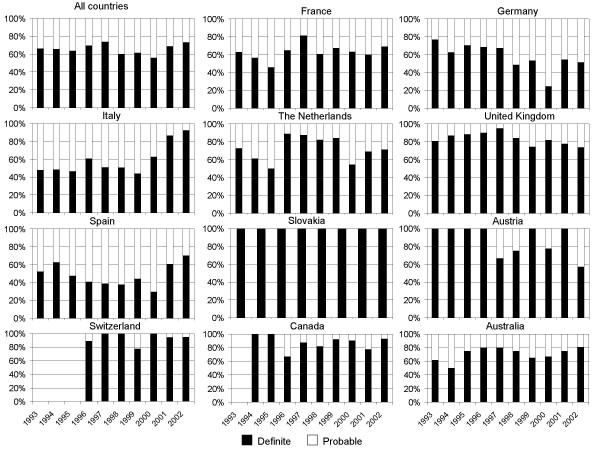
Annual proportion of histopathologically-confirmed, i.e. definite, sporadic CJD in EUROCJD countries.

Annual histopathological confirmation of sCJD (definite cases) is depicted in Figure 3. There was considerable variation between individual countries, with proportions of definite sCJD varying from 100% in Slovakia to as low as 61% in France, 59% in Italy, 57% in Germany and 52% in Spain. Noteworthy was the annual variation in diagnostic confirmation of sCJD statistically significant, both overall, p < 0.001, and for specific countries, such as France, p = 0.007, Germany, p < 0.001, Spain p = 0.002, UK p = 0.038 and Austria (Fisher's exact test, p = 0.019). When comparing national, definite versus probable confirmation ratios, statistically significant low values were found for Spain (OR 0.50, 95%CI 0.41–0.62), Germany (OR 0.60 95%0.52–0.70), Italy (OR 0.68 95%CI 0.58–0.81), and France (OR 0.75 95%CI 0.65–0.88), with no differences in time trend being identified for the four countries (comparative time trend OR 1.00 95%CI 0.98–1.03).

**Figure 4:**
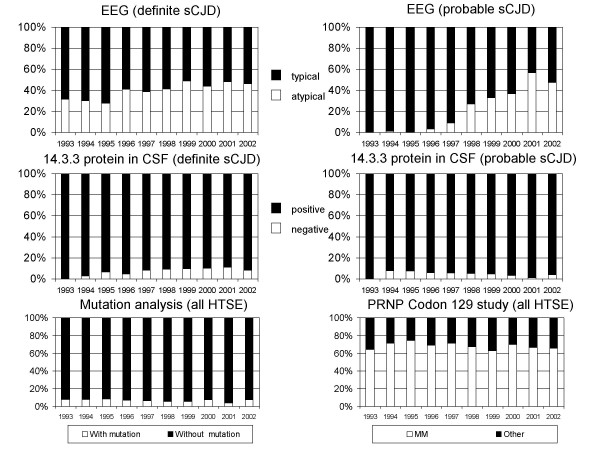
Annual distribution of positive results in EEG and 14.3.3 protein tests in sCJD, and for all HTSE, PRNP mutations identified (in patients who had and had not been genetically studied) and PRNP codon 129 genotype.

The yearly distribution patterns of diagnostic tests -such as 14.3.3 protein detection in CSF, EEG and genetic analyses in all HTSE- are shown for all countries in Figure 4. During the study period, the proportions of definite and probable sCJD deaths with available EEG results in which PSWCS were identified were 58% and 73% respectively. The annual variations proved statistically significant, p < 0.001 in both cases, suggesting a rising time trend in atypical EEG patterns in sCJD since 1997. The proportions of definite and probable sCJD with 14.3.3 tests were 62% and 74% respectively for the whole period; annual variation was not statistically significant, with 88% and 95% of such tests proving positive. Genetic analyses were performed on 64% of all HTSE. Mutations could be detected in 8.5% of all HTSE (7.04% missense mutations, 1.04% insert mutations). The annual proportion of methionine homozygous cases in *PRNP *129 codon for all genetically examined HTSE patients did not vary significantly, p = 0.116.

The annual proportions of probable sCJD cases having exclusively positive EEG or 14.3.3 test results, along with the proportion of gHTSE identified by genetic analysis only (without family history of prion diseases) are listed in Table 2. The annual proportion of probable sCJD solely having a positive EEG declined evenly with time, from 95% to 3% across the period. Up to 1998, few probable sCJD cases solely had a positive 14.3.3 test (diagnostic criteria changed in 1998), but in that year the annual number rose sharply, after which it fell slightly to remain at a mean proportion of 46%. The annual proportion of gHTSE identified by mutation alone was fairly stable over the study period, with a mean of 51%.

## Discussion

In terms of case numbers, this study constitutes the largest-scale observation of HTSE ever conducted. Prior reports on this clinical population are sparse [10]. Taking into account that neither mortality of HTSE nor population denominators are considered in this study, the results suggest that: 1) the panorama of HTSE, as seen from deaths for all or part of the 1993–2002 period in 11 countries, varies within and between countries, sometimes exhibiting characteristic features; 2) there is an expected, overall rising time trend in annual deaths and proportions of patients studied using ancillary tests other than EEG; 3) characteristic national patterns, as seen from the magnitude of and time-trends for proportions of specific entities, were particularly relevant in the UK and France for vCJD and iCJD, and in Slovakia and Italy for gHTSE; 4) pathological confirmation of sCJD varied but international differences persisted across time; and, 5) the additional contribution of ancillary tests to sCJD diagnosis decreased to almost nil for EEG, increased to stable figures for the 14.3.3 test, and was high and stable across time for genetic assay insofar as gHTSE was concerned. The interpretation of these results is complicated by several parameters, due to the fact that use of methods for diagnosing HTSE, particularly CSF 14.3.3 test and MRI, improved significantly over the course of the study period.

The eligibility of cases for this study based on vital status after death proved most appropriate, since diagnostic criteria for probable sCJD may require measurement of disease duration, <2 years, and quality of diagnosis is frequently determined by post-mortem examination. Diagnosis classification for probable cases is therefore neither conditional to a specific disease course nor provisory. A rising time-trend in sCJD incidence or mortality has been observed over the last decade in Austria, France, Germany, Italy, Switzerland, the UK, and other countries [9,23-25] and has mainly been attributed to progressive, persistent, improvement in sCJD diagnostic ascertainment [26, 27] or has gone unexplained [9]. This large dataset makes the EUROCJD countries the most stable reference population for comparing HTSE incidence, e.g., that of sCJD in specific age-groups. Nonetheless, undercounts due to poor reporting or case ascertainment before 1998, likely due to different awareness and clinical management of dementia prior to 14.3.3 CSF test, and incomplete case-finding after 2001 by observation at death, suggest that the optimal time interval for incidence measurements using this material should be carefully selected, e.g., 2000–2001 for sCJD.

With regard to changes across time, differences due to the presence of vCJD in the UK and France are most remarkable. Abrupt changes in 1997 and 1998 for sCJD suggest a strong impact of the first vCJD report [4] on diagnostic practices and the updating of diagnostic criteria. Yet, interpretation of comparative linear time trends can sometimes prove problematic. For instance, changes in average age at death might reflect improved ascertainment for sCJD and an exposure-related cohort effect with increasing duration of incubation period for iCJD.

Changes across time reported here may, among other things, reflect clinical management and reporting, and variations in pre- and post-mortem laboratory diagnostic practices and diagnostic criteria updates. The increasing use of MRI might explain rising trends for CJD since specific patterns of abnormality on diffusion-weighted and fluid-attenuated inversion recovery images are highly sensitive and specific for CJD [28]. In EUROCJD countries, the use by clinicians of the 14.3.3 protein test in sCJD diagnosis first became significant in 1997. Accordingly, the 14.3.3 protein in CSF, identified for the not inconsiderable annual proportions of HTSE patients who died from 1993 to 1996 (Figure 1, bottom right) -with test results shown for definite and probable sCJD (Figure 4, centre) and probable sCJD (Table 2)- should have been determined post-mortem, in most cases on frozen CSF stored for research purposes. This policy might have had: a) positive effects, in terms of increasing the average amount of information per registered HTSE case, something that is particularly interesting for definite sCJD cases with date of death prior to 1998; and b) bias in research, due to inclusion of 14.3.3-test results in the database for probable sCJD being made conditional upon the presence of PSWCs in EEG, a requisite for such diagnosis before 1998. The study of associations similar to those reported [29, 30, 31] using this large database may benefit from stratification by period of death or diagnostic criteria for probable sCJD.

Identifying a time-related increase in sCJD incidence in populations with valine in PRNP codon 129 which have been considerably exposed to BSE has been a key goal of EUROCJD-sponsored public health HTSE surveillance, reinforced by results of recent laboratory research [15,16]. The frequent lack of PRNP codon 129 data for age-specific national populations makes it impossible to calculate the incidence figures required as denominators for such analyses. Hence, a surrogate index is currently used, comparing proportions of sCJD with a valine allele in the UK versus those in other countries and their changes across time [31]. Our study results for the general population suggest that there have been no changes over time in this proportion since 1996 (Figure 4, bottom right). Due to frequent atypical clinical features [32], probable sCJD is particularly liable to being misdiagnosed in patients with valine in codon 129, and as a result expected improvement in sCJD diagnosis internationally is not reflected as a trend. Monitoring international changes across time in clinical-geno-phenotype with this extensive and unique material might require stratification by variables potentially associated with misdiagnosis and codon 129 structure, e.g., EEG pattern.

## Conclusion

This study reveals remarkable international differences in the HTSE panorama that change with time, as seen from deaths in eleven countries in the period 1993–2002. Knowledge of possible biases in the study cohort is vital for future applications of this dataset, both in clinical/epidemiological research and in public health surveillance.

## Competing interests

The author(s) declare that they have no competing interests.

## Authors' contributions

The EUROCJD consortium generated the EUROCJD Research Database which constituted the dataset used in this study. JP contributed with study design, design and supervision of analysis and drafted first manuscript. MG and KS reviewed and drafted biologically relevant paragraphs. JA did the statistical analysis. VM, MP and MP contributed to the data base refinement and classification of genetic forms. All other authors, particularly and repeatedly AA, CvD, SC, VL, GHJ and MC, contributed with comments to several versions and criticisms of methods and text, AA, CvD, SC, VL and figures AA, GHJ. All authors read and approved the final manuscript.

## Pre-publication history

The pre-publication history for this paper can be accessed here:



## Figures and Tables

**Table 1:** Number of cases studied by diagnostic entity and country

	Sporadic CJD	Variant CJD	Iatrogenic CJD	Genetic TSE				All categories
					
				CJD	GSS	FFI	Insert	
Australia	189	-	4	14	4	3	1	215
Austria*	77	-	-	9	-	3	1	90
Canada**	169	1	3	7	9	-	-	189
France	766	6	82	68	5	6	5	938
Germany	827	-	5	31	8	17	12	900
Italy	544	-	3	94	8	10	3	662
Netherlands	136	-	3	1	-	-	2	142
Slovakia	18	-	-	41	-	-	-	59
Spain*	380	-	5	18	-	25	1	429
Switzerland	84	-	-	1	-	-	-	85
UK	530	121	33	11	19	1	17	732
All countries	3720	128	138	295	53	65	42	4441

* Since 1996, including retrospective cases from 1993. ** Since 1998, including retrospective cases from 1994

**Table 2:** Additional contribution of: positive results in EEG and CSF examination (14.3.3 protein test) to diagnosis of probable sporadic CJD; and identified PrP gene mutation to diagnosis of genetic HTSE.

Year of death	Cases solely with positive EEG Number and percentage of probable sporadic CJD cases	Cases solely with positive CSF 14.3.3 test Number and percentage of probable sporadic CJD cases	Cases with mutation and negative or missing data on family history. Number and percentage of genetic TSE cases
1993	52 (95)	-	13 (48)
1994	60 (70)	7 (8)	16 (50)
1995	61 (68)	4 (4)	15 (45)
1996	62 (64)	13 (14)	23 (55)
1997	30 (30)	21 (21)	20 (53)
1998	32 (18)	65 (36)	26 (55)
1999	15 (9)	67 (38)	24 (49)
2000	11 (6)	79 (41)	23 (40)
2001	8 (5)	95 (58)	30 (44)
2002	4 (3)	69 (54)	40 (65)
